# Successful Limb Salvage in a Diabetic Septic Foot Using Anterolateral Thigh Free Flap Reconstruction: A Case Report

**DOI:** 10.7759/cureus.108422

**Published:** 2026-05-07

**Authors:** Nay Aung Zin, Thura Kyaw, Cherry Myint, Yin W Thant, Shoon L Lwin, Tun Aung Khine, Nang Hsu Yi Htwe

**Affiliations:** 1 Orthopaedics and Traumatology, Kulhudhuffushi Regional Hospital, Kulhudhuffushi, MDV; 2 Orthopaedics and Trauma, Yangon Orthopaedic Hospital, Yangon, MMR; 3 Orthopaedics and Trauma, 300 Bedded Orthopaedic Hospital, Mandalay, MMR; 4 Emergency Medicine, Kulhudhuffushi Regional Hospital, Kulhudhuffushi, MDV; 5 Dermatology, Medica Hospital, Malé, MDV; 6 Medicine and Surgery, Makunudhoo Health Center, Makunudhoo, MDV; 7 Emergency Medicine, Indira Gandhi Memorial Hospital, Malé, MDV; 8 General Practice, Damaw Thada Social Clinic, Yangon, MMR

**Keywords:** anterolateral thigh flap, diabetic foot, free flap, limb salvage, microsurgery, septic wound, soft tissue reconstruction

## Abstract

Severe soft tissue defects of the foot in patients with diabetes mellitus complicated by septicemia pose a significant reconstructive challenge and are frequently associated with a high risk of amputation. Limb salvage in such cases requires aggressive infection control, meticulous debridement, and reliable soft tissue coverage.

We report the case of a 57-year-old man with poorly controlled diabetes who developed an extensive dorsal foot defect following trauma, complicated by infection. After serial debridement and optimization of systemic condition, reconstruction was performed using an anterolateral thigh free flap. Microvascular anastomosis was successfully achieved using the dorsalis pedis artery and venae comitantes.

The postoperative course was uneventful, with complete flap survival and no recurrence of infection. At the six-month follow-up, both donor and recipient sites were well healed, and the patient regained functional ambulation. Although the flap appeared cosmetically bulky, it provided durable coverage and prevented amputation.

This case highlights the effectiveness of an anterolateral thigh free flap in achieving limb salvage in high-risk diabetic patients, emphasizing that functional preservation should take precedence over cosmetic outcomes.

## Introduction

Soft tissue defects of the foot in patients with diabetes mellitus represent a significant clinical challenge due to impaired wound healing, increased susceptibility to infection, and associated vascular compromise. Diabetic foot complications are a leading cause of non-traumatic lower limb amputation worldwide, and failure to achieve adequate soft tissue coverage is strongly associated with poor functional outcomes and increased mortality. These factors highlight the importance of timely and effective reconstructive strategies aimed at limb preservation.

Free tissue transfer has significantly advanced the management of complex soft tissue defects. A free flap involves the transfer of well-vascularized tissue from a distant donor site to the defect, with restoration of blood supply through microvascular anastomosis. Among these, the anterolateral thigh (ALT) flap, first described by Song et al. [1], has become a widely utilized option due to its reliable vascular anatomy and versatility. Subsequent large clinical series have demonstrated its consistent success and adaptability across a broad range of reconstructive applications [2,3].

In diabetic patients, limb salvage remains the primary objective, as amputation is associated with substantial morbidity, mortality, and reduced quality of life [4]. In addition, vascular compromise plays a key role in delayed wound healing, and appropriate assessment of perfusion is essential in planning reconstruction [5]. While free flap reconstruction is well established in traumatic lower limb defects, its application in chronically infected diabetic wounds presents additional challenges.

Previous studies, such as that by Yazar et al. [6], have demonstrated the effectiveness of free flap reconstruction in traumatic composite defects, while Oh et al. [7] emphasized the benefits of early microsurgical reconstruction. However, the present case differs in that reconstruction was delayed due to the need for infection control in a septic diabetic wound. Despite this delay, successful reconstruction was achieved, highlighting the importance of appropriate timing based on wound condition rather than fixed surgical principles.

The ALT flap offers several advantages in lower limb reconstruction, including the ability to provide a large skin paddle, reliable vascularity, and flexibility in design [8]. However, reports specifically addressing its use in high-risk diabetic patients with prolonged wound chronicity and systemic infection remain limited.

We present a case of successful limb salvage in a patient with poorly controlled diabetes (HbA1c 9.8%), septic presentation, and a four-month history of wound chronicity, managed using an ALT free flap. This case highlights the feasibility of free flap reconstruction in a high-risk setting and emphasizes the importance of infection control, careful patient selection, and prioritization of functional outcomes.

## Case presentation

A 57-year-old man with a 15-year history of poorly controlled diabetes mellitus (HbA1c: 9.8%) presented with a non-healing wound over the dorsum of the left foot following a road traffic accident four months prior. During this interval, the patient received intermittent wound care and oral antibiotics at a local facility without definitive surgical management, resulting in progressive wound deterioration.

On presentation, the patient exhibited clinical features consistent with sepsis, including fever (38.5°C), tachycardia (110 beats/min), and elevated inflammatory markers (white blood cell count and C-reactive protein). There was no hypotension. Blood culture data were not available.

**Figure 1:**
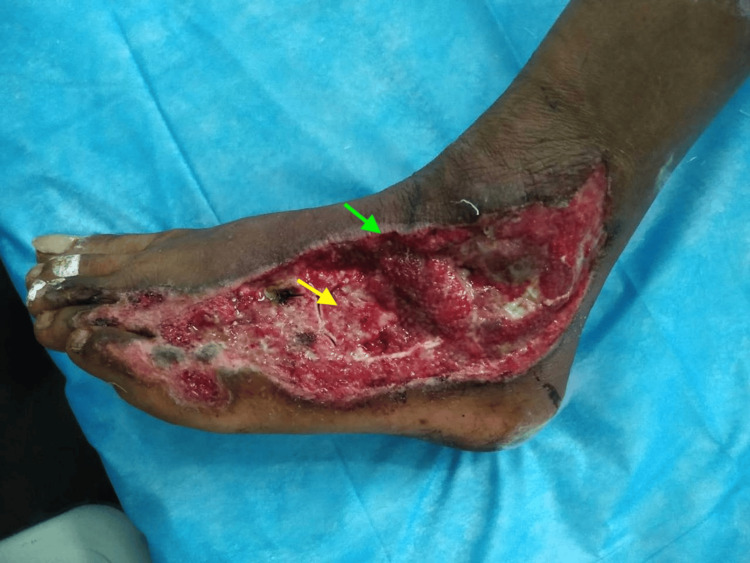
Preoperative dorsal foot defect Extensive soft tissue defect over the dorsum of the left foot measuring approximately 14×7 cm. The yellow arrow indicates the exposed bone within the wound bed, likely involving the midfoot (cuneiform region). The green arrow highlights the surrounding inflamed and necrotic soft tissue. The wound demonstrates features of severe infection with irregular margins and non-viable tissue.

On examination, there was an extensive soft tissue defect measuring approximately 14×7 cm over the dorsal aspect of the left foot, with exposed extensor tendons, tarsal bones, and surrounding cellulitis (Figure 1). The wound bed contained slough and necrotic tissue.

The patient underwent serial surgical debridement on three occasions over a two-week period to achieve adequate source control. The procedures were performed at approximately 4-5-day intervals, allowing the reassessment of tissue viability between stages. Each debridement involved thorough excision of necrotic skin, subcutaneous tissue, and devitalized fascia, with careful preservation of viable extensor tendons where possible. The wound was irrigated with copious normal saline, and hemostasis was secured at each stage.

Negative pressure wound therapy (NPWT) was applied between debridements to promote granulation tissue formation, reduce wound edema, and assist in infection control. The wound was reassessed clinically at each stage for reduction in slough, absence of purulence, and improvement in surrounding soft tissue condition.

Empirical broad-spectrum intravenous antibiotics (ceftriaxone and metronidazole) were initiated at presentation and continued based on clinical response and improvement in inflammatory markers. Wound cultures were obtained after the initiation of antibiotics and demonstrated no bacterial growth, likely reflecting prior antimicrobial therapy.

Definitive reconstruction was planned once the wound demonstrated a clean granulating bed, absence of clinical signs of infection, and normalization of inflammatory markers.

Strict glycemic control was achieved using an insulin-based regimen, targeting blood glucose levels between 140 and 180 mg/dL during hospitalization.

**Figure 2:**
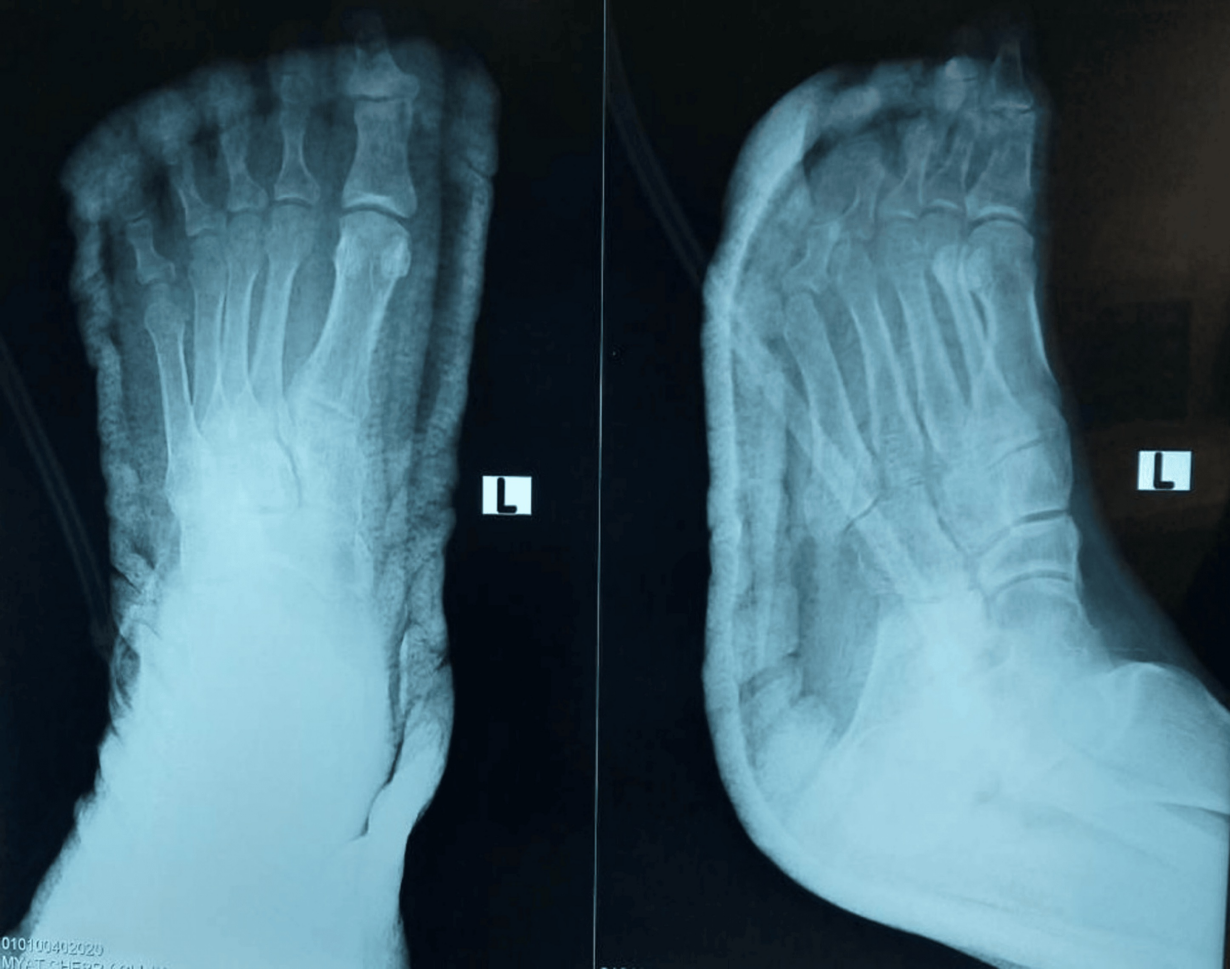
Radiographic evaluation No radiographic features suggestive of osteomyelitis (noting the limited sensitivity of plain radiographs in early disease).

Following infection control, laboratory investigations including complete blood count, erythrocyte sedimentation rate, and C-reactive protein normalized. Plain radiographs of the left foot showed no evidence of osteomyelitis (Figure 2).

Peripheral vascular assessment revealed palpable dorsalis pedis and posterior tibial pulses. Due to resource limitations, advanced vascular imaging such as computed tomography (CT) angiography was not performed. However, adequate intraoperative arterial flow was confirmed prior to microvascular anastomosis.

After optimization of the patient's condition and adequate wound bed preparation, definitive reconstruction was planned using an ALT free flap.

**Figure 3:**
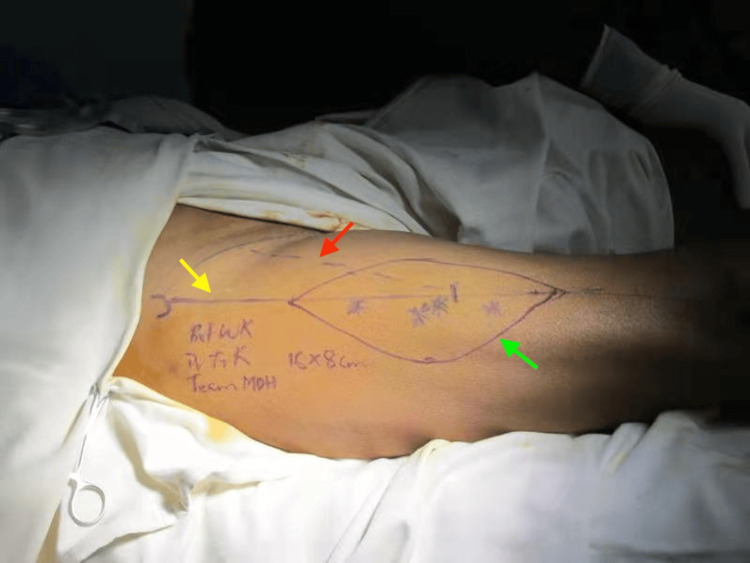
Flap planning Skin markings demonstrate the planned flap design (16×8 cm) centered along the axis between the anterior superior iliac spine and the lateral border of the patella (yellow arrow). The red arrow indicates the central perforator location used for flap design, while the green arrow outlines the distal margin of the skin paddle.

Preoperative Doppler assessment was used to identify suitable perforators. A fasciocutaneous flap measuring approximately 16×8 cm was designed over the ALT (Figure 3), slightly oversized relative to the defect to allow for tension-free inset and coverage of the surrounding compromised tissue.

**Figure 4:**
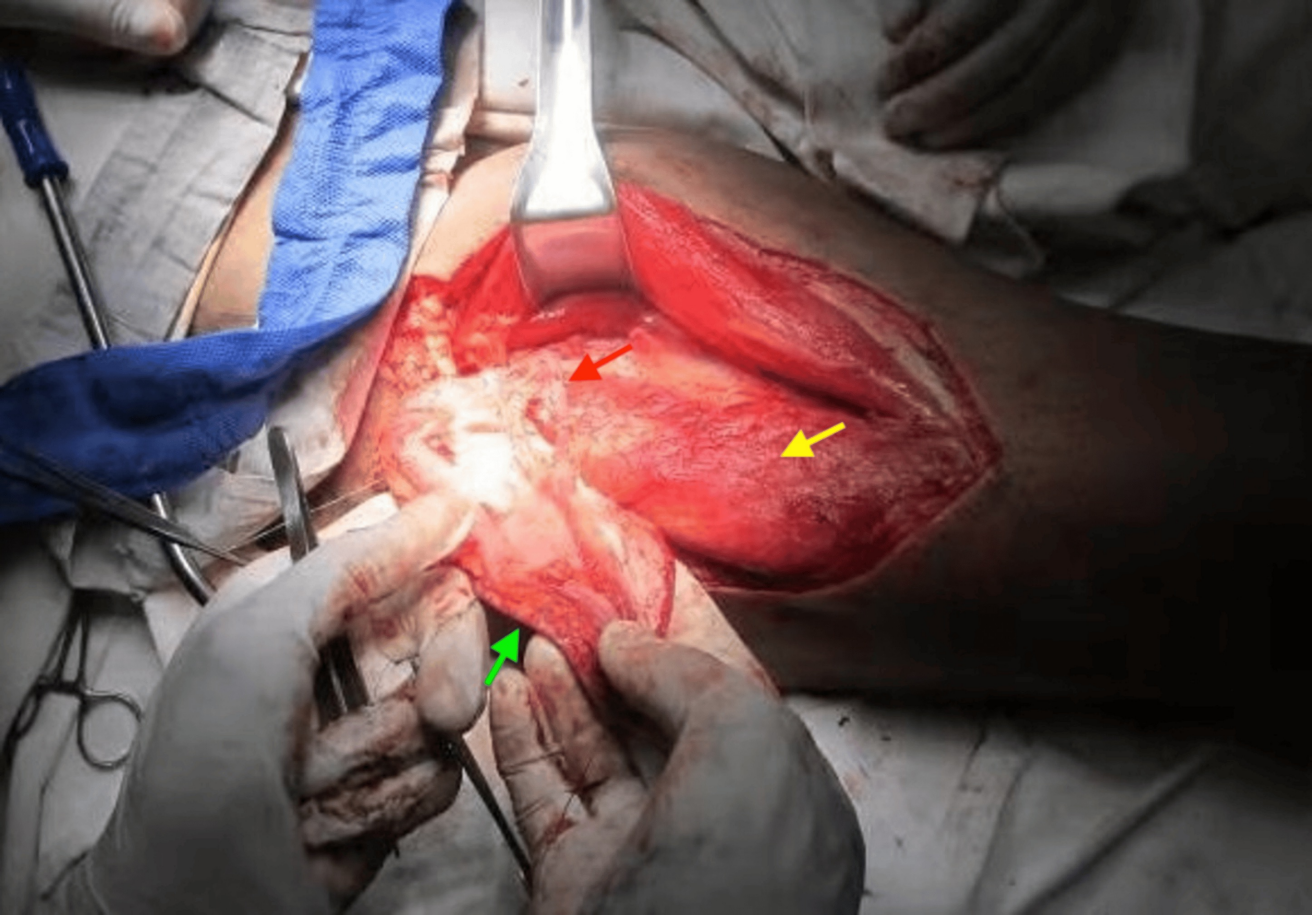
Intraoperative elevation of the anterolateral thigh flap Dissection of the flap reveals a septocutaneous perforator (red arrow) arising from the descending branch of the lateral circumflex femoral artery. The yellow arrow indicates the vastus lateralis muscle, while the green arrow highlights the vascular pedicle.

**Figure 5:**
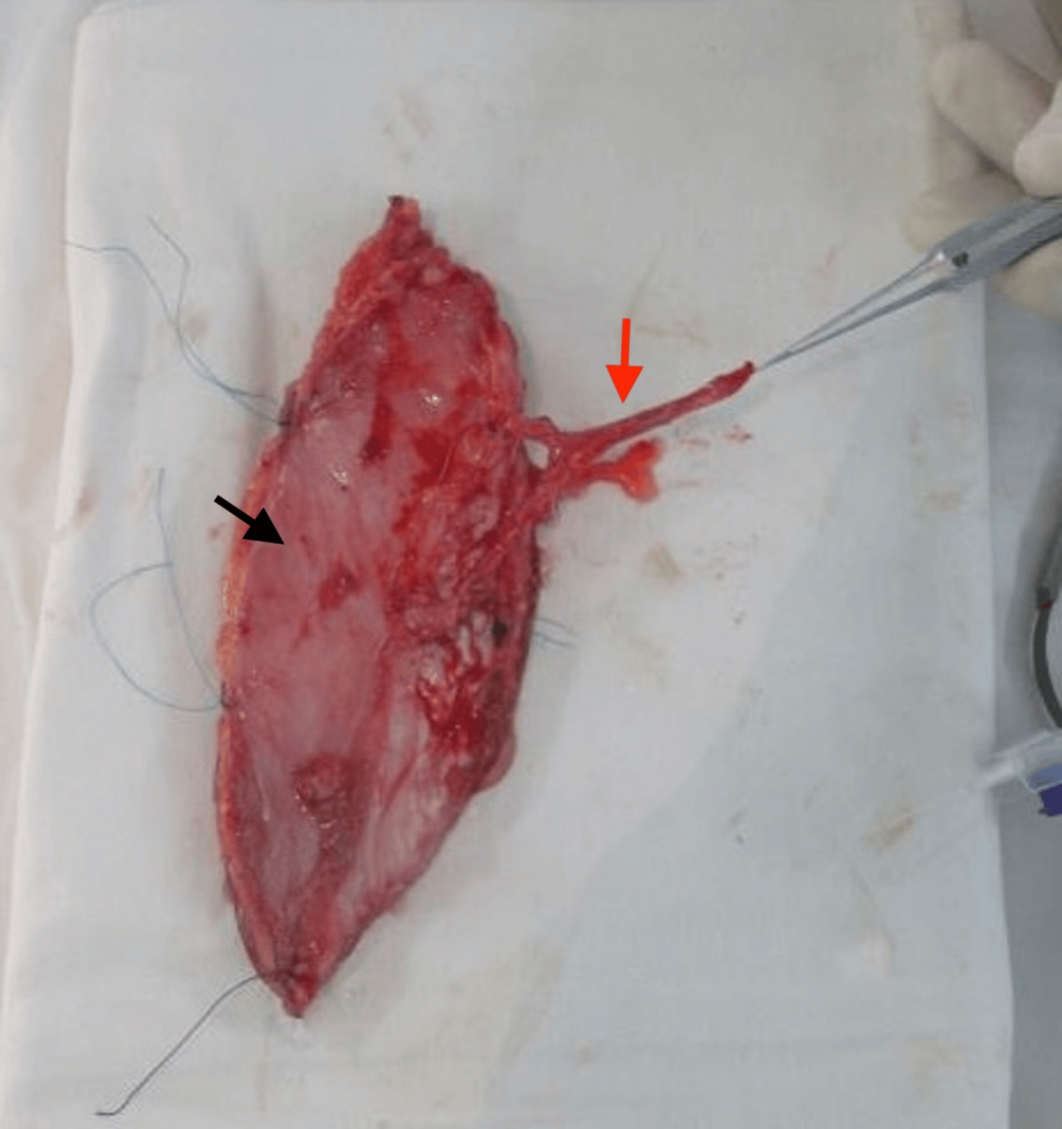
Harvested anterolateral thigh free flap The harvested flap demonstrates a well-vascularized skin paddle (black arrow) with its vascular pedicle (red arrow), consisting of one artery and accompanying veins, prepared for microvascular transfer.

The flap was raised based on a musculocutaneous perforator arising from the descending branch of the lateral circumflex femoral artery. The vascular pedicle length was approximately 7 cm (Figures 4-5). Although shorter than typically described, this length was sufficient due to the proximity of recipient vessels and did not pose a technical difficulty.

At the recipient site, the dorsalis pedis artery and its venae comitantes were dissected and prepared. Microvascular anastomosis was performed in an end-to-end fashion between the flap artery and the dorsalis pedis artery using interrupted 9-0 nylon sutures under an operating microscope.

Venous drainage was established using two venous anastomoses. One vena comitans was anastomosed using standard hand-sewn technique with 9-0 nylon sutures, while the second vein was anastomosed using a 2.5 mm venous coupler device. The total flap ischemia time was approximately 75 minutes.

**Figure 6:**
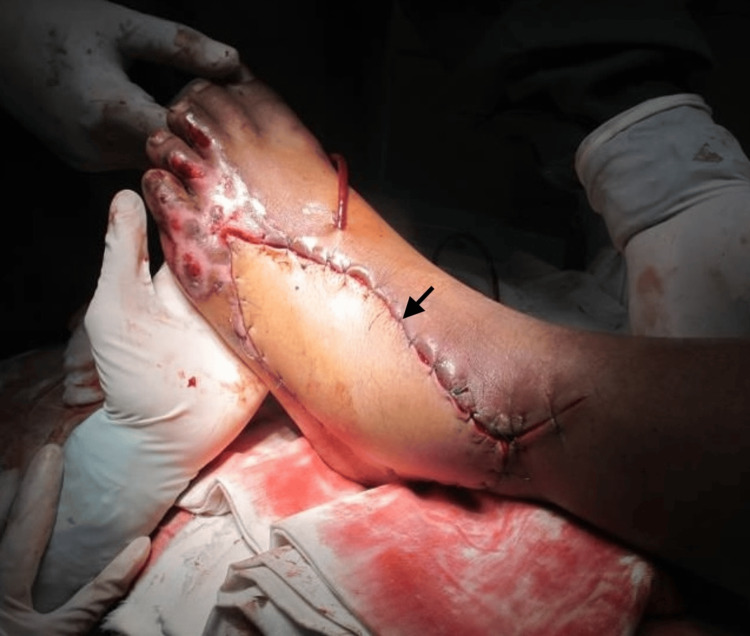
Inset of the anterolateral thigh flap at the recipient site The flap is inset over the dorsal foot defect following microvascular anastomosis. The black arrow indicates the flap margin and suture line, demonstrating adequate coverage of the previously exposed extensor tendons.

Adequate perfusion was confirmed intraoperatively prior to inset, and the flap was inset without tension to achieve complete coverage of the defect (Figure 6). The donor site was closed primarily.

Postoperatively, the patient was closely monitored for flap viability, including hourly assessment during the first 24 hours, followed by regular monitoring. Limb elevation and immobilization were maintained. Meticulous wound care was emphasized, including aseptic dressing changes, infection surveillance, and strict glycemic control.

At the six-week follow-up, the flap remained viable with satisfactory wound coverage. At three months, stable soft tissue coverage was maintained, and the patient was able to ambulate with protective footwear. At six months, both donor and recipient sites were well healed.

The patient regained independent ambulation for daily activities with protective footwear. No ulcer recurrence or flap compromise was observed. There was no significant donor site morbidity, and the patient did not report thigh weakness or sensory disturbance.

**Figure 7:**
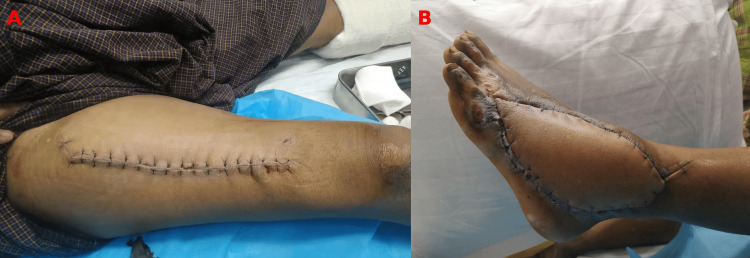
Final outcome (A) Donor site following primary closure with satisfactory healing. (B) Recipient site showing well-healed anterolateral thigh flap with stable soft tissue coverage; note mild bulkiness over the dorsum of the foot.

Although the flap appeared cosmetically bulky (Figure 7A-7B), it provided durable and stable coverage without compromising function. The patient was counseled regarding secondary debulking; however, he declined further intervention as the bulk did not significantly interfere with daily activities.

A summary of the clinical course and key management steps is presented in Table 1.

**Table 1:** Clinical timeline of patient management

Time point	Clinical event	Key findings/management
Month 0 (injury)	Road traffic accident	Soft tissue injury over the dorsum of the left foot
Months 0-4	Initial management at the local facility	Intermittent wound care and oral antibiotics, no definitive surgical intervention
Presentation (month 4)	Admission to hospital	14×7 cm dorsal foot defect with exposed extensor tendons, signs of infection and sepsis
Days 1-14	Serial debridement (3 sessions)	Removal of necrotic tissue; wound bed preparation, IV antibiotics initiated
During admission	Medical optimization	Insulin-based glycemic control (target 140-180 mg/dL); infection monitoring
Post-debridement	Reassessment	Normalized inflammatory markers, no radiographic evidence of osteomyelitis
Surgery	Anterolateral thigh free flap reconstruction	16×8 cm flap; 4 cm pedicle, microvascular anastomosis, ischemia time -75 minutes
Postoperative days 1-7	Flap monitoring	Hourly monitoring initially, stable flap perfusion
6 weeks follow-up	Early outcome	Viable flap, satisfactory wound coverage
3 months follow-up	Functional outcome	Independent ambulation with protective footwear
6 months follow-up	Final outcome	Well-healed donor and recipient sites, no recurrence; bulky but functional flap

## Discussion

The ALT flap has evolved into a workhorse flap in reconstructive microsurgery since its initial description by Song et al. [1]. Its versatility and consistent vascular anatomy have been validated by large clinical series, including those by Wei et al. [2] and Kuo et al. [3], which demonstrated its reliability in a wide range of soft tissue reconstructions.

In diabetic patients, management of complex foot defects is particularly challenging due to impaired wound healing, neuropathy, and increased susceptibility to infection. The primary objective in such cases is the preservation of limb function, as amputation is associated with significant morbidity and decreased quality of life [4]. In addition, vascular compromise plays a critical role in delayed healing, and assessment of perfusion is essential in determining reconstructive options [5].

In the present case, vascular assessment was limited to clinical examination due to resource constraints, but adequate perfusion was confirmed intraoperatively. Despite the absence of advanced imaging, successful microvascular anastomosis and flap survival were achieved, highlighting that careful intraoperative assessment can be sufficient in selected settings.

Free tissue transfer is often required when local or regional flaps are insufficient to cover large or complex defects. Yazar et al. [6] demonstrated the effectiveness of free flap reconstruction in traumatic composite lower extremity defects, highlighting its role in restoring both soft tissue coverage and function. However, the present case involved a chronic infected diabetic foot wound, which represents a different reconstructive challenge. More directly applicable evidence suggests that free flap reconstruction can support limb salvage in selected diabetic patients [7]. In our case, the size of the defect and exposure of extensor tendons precluded the use of local or regional flaps, making free tissue transfer the most appropriate reconstructive option.

Early reconstruction has been associated with improved outcomes in complex extremity injuries, as described by Godina [8]. However, in this patient, reconstruction was delayed due to ongoing infection. Adequate debridement and infection control were prioritized before definitive reconstruction, resulting in a favorable outcome. This supports the principle that, although early reconstruction is ideal, delayed reconstruction can still be successful when infection is properly managed.

The ALT flap offers several advantages, including the ability to provide a large skin paddle with reliable vascularity and minimal donor site morbidity. Wong and Wei [9] emphasized its role as a versatile flap suitable for complex defects. In the present case, the flap provided durable coverage and facilitated limb salvage in a high-risk patient.

The relatively short pedicle length in this case did not compromise the outcome due to the proximity of recipient vessels. Additionally, the use of dual venous anastomoses likely contributed to reliable venous drainage and flap survival.

Although the flap appeared cosmetically bulky, this is an acceptable limitation in limb salvage procedures. The primary objective is stable wound coverage and preservation of function, with secondary debulking procedures available if required. In this case, the patient declined further surgery as the bulk did not significantly affect daily activities.

Meticulous postoperative care, including strict glycemic control and regular wound monitoring, played a crucial role in achieving a successful outcome. This highlights the importance of a multidisciplinary approach in managing complex diabetic foot defects.

## Conclusions

This case demonstrates that an ALT free flap can be an effective reconstructive option for extensive dorsal foot defects in patients with diabetes complicated by infection. Successful limb salvage in this setting depends on adequate infection control, thorough wound bed preparation, and careful selection of reconstructive technique.

Although the flap resulted in a cosmetically bulky appearance, it provided durable soft tissue coverage and allowed the restoration of functional ambulation. This highlights that, in complex diabetic foot reconstruction, preservation of limb function and prevention of amputation should take priority over aesthetic considerations.

This report describes a single case and therefore has limited generalizability. The follow-up period of six months is relatively short to assess long-term durability under weight-bearing conditions. Objective functional outcome measures were not utilized. Additionally, advanced vascular imaging was not performed due to resource limitations. Further studies with larger patient cohorts and longer follow-up are required.
